# Plant-derived, stress-responsive microRNA mimics reduce powdery mildew infections of barley

**DOI:** 10.3389/fpls.2026.1885955

**Published:** 2026-08-27

**Authors:** Aftab Ahmad, Kinza Khalid, Alexander Idnurm, Niloofar Vaghefi, Levente Kiss

**Affiliations:** 1 Centre for Crop Health, School of Science, Engineering and Digital Technologies, University of Southern Queensland, Toowoomba, QLD, Australia; 2 School of BioSciences, Faculty of Science, The University of Melbourne, Melbourne, VIC, Australia; 3 School of Agriculture, Food and Ecosystem Sciences, Faculty of Science, The University of Melbourne, Melbourne, VIC, Australia; 4 Food and Wine Research Centre, Eszterházy Károly Catholic University, Eger, Hungary; 5 Plant Protection Institute, HUN-REN Centre for Agricultural Research, Budapest, Hungary

**Keywords:** *Blumeria hordei*, cross-kingdom targets, micro-colony index, miRNA mimics, miRNA duplexes, spray-induced gene silencing, (SIGS), stress responsive miRNAs

## Abstract

Barley powdery mildew, caused by *Blumeria hordei*, leads to economic losses worldwide. Here, *in silico* analyses and proof-of-concept experiments were conducted to determine if specific plant-derived synthetic microRNAs (miRNAs) may be considered as a new direction for the control of barley powdery mildew. Previous studies have demonstrated that barley miRNAs regulate plant immunity against *B. hordei*. Based on the predicted targets of stress-responsive miRNAs in barley, four miRNAs that are upregulated during biotic stresses were selected and applied through Spray-Induced Gene Silencing (SIGS). Double-stranded RNA (dsRNA) constructs were designed and synthesized for the four selected miRNAs and sprayed on detached barley leaf segments that were subsequently infected with *B. hordei*. Visual disease symptoms and the *B. hordei* micro-colony index were significantly reduced in the treated leaf segments compared to the control. Quantitative PCR analysis confirmed a significant reduction in the relative *B. hordei* DNA abundance six days post infection. These results confirmed that the selected plant-derived synthetic miRNAs suppress the development of barley powdery mildew under experimental conditions. This is the first study to show that plant-derived, stress-responsive synthetic miRNAs reduced infection caused by fungal pathogens when applied through SIGS. Our approach could be applied in further experiments focusing on functional analyses of miRNAs in a time-efficient manner, and without requiring miRNA overexpression via genetic transformation.

## 1 Introduction

RNA interference (RNAi) is a regulatory mechanism in which small RNAs (sRNAs) inhibit gene expression at the transcriptional or post-transcriptional level by neutralizing target messenger RNAs (mRNAs). Additionally, RNAi contributes to genome stability by controlling transposon activity (Krzyszton et al., 2025). Core components of the RNAi pathways such as Dicer-like proteins (DCLs), Argonaute proteins (AGOs), and RNA-dependent RNA polymerases (RDRs) are evolutionarily conserved across eukaryotic organisms (Cao et al., 2016; Cui et al., 2020; Wang et al., 2024).

In some cases, sRNAs are produced to induce gene silencing in organisms interacting with the source, even when those organisms belong to a different kingdom. Well-studied examples of cross-kingdom RNA interference (CKRI) include interactions between plants and their pathogens belonging to diverse kingdoms of microorganisms (Zhang et al., 2016; Cai et al., 2018; Kusch et al., 2018, 2023; Mueth and Hulbert, 2022; Shahid et al., 2018; Wang M. et al., 2016; Weiberg et al., 2013). Some studies consider CKRI as a third layer of plant immunity, complementing pattern-triggered and effector-triggered immune responses (Conrath, 2025; Zhang et al., 2016). Plants produce sRNAs derived from their transposable elements (TEs) and resistance (R) genes, which not only regulate the functioning of these elements within their tissues but can also be transferred to their pathogens and target specific genes to suppress the pathogens’ virulence (Mahanty et al., 2023; Shikha et al., 2025; Weiberg et al., 2014; Zhang et al., 2016). CKRI focused on the interactions between *Arabidopsis* and its fungal pathogen *Botrytis cinerea* revealed that *Arabidopsis* plants secrete exosome-like extracellular vesicles (EVs) that transfer two sRNAs into the pathogen (Cai et al., 2018). These sRNA-containing vesicles gather at the infection sites and are transferred to the fungal hyphae. The two specific sRNAs produced by *Arabidopsis* target *B. cinerea* genes that are involved in vesicle trafficking pathways and pathogenicity of the fungal pathogen (Cai et al., 2018).

Plant sRNA-induced suppression of pathogen genes is not limited to fungal pathogens. For example, it was documented in interactions between *Arabidopsis* and its oomycete pathogen *Phytophthora capsici* (Hou et al., 2019) and its bacterial pathogens, as well, including *Pseudomonas syringae* (Singla-Rastogi et al., 2019).

Some fungal plant pathogens can uptake specific, plant-derived double-stranded RNAs (dsRNAs) from the environment that initiate gene silencing through endogenous RNAi pathways within the target fungi (Koch et al., 2016; McRae et al., 2023; Qiao et al., 2021; Ouyang et al., 2025; Wang M. et al., 2016). This discovery paved the way for bioengineered applications of CKRI, for example through Spray-Induced Gene Silencing (SIGS) (Mahanty et al., 2023). SIGS was successfully applied in experiments targeting interactions between diverse crops and their fungal plant pathogens (Bocos-Asenjo et al., 2025; Gu et al., 2019; He et al., 2024; Koch et al., 2016; McLoughlin et al., 2018; Song et al., 2018; Wang M. et al., 2016). The application of synthetic dsRNAs that control crop pathogens through SIGS has been considered as a promising and environmentally friendly alternative to crop protection based on fungicide applications (Chen X. et al., 2025; Hough et al., 2022; Mosquera et al., 2025; Zhao et al., 2024).

Powdery mildew of barley (*Hordeum vulgare*), caused by *Blumeria hordei* (Liu M. et al., 2021), is an economically significant disease wherever barley is grown (Murray and Brennan, 2010; Singh, 2017; Ellwood et al., 2024). Powdery mildew can lead to moderate to severe grain yield losses in barley: under favorable disease conditions and with susceptible varieties, losses of 12% to 60% may occur (Ellwood et al., 2024). Currently, its management is largely based on growing resistant or tolerant cultivars and application of synthetic fungicides (Dreiseitl, 2023; Ellwood et al., 2024). Research on new disease management options is supported due to the increasing prevalence of *B. hordei* resistance to synthetic fungicides (Tucker et al., 2020; Vielba-Fernández et al., 2020; Zulak et al., 2018), increasing restrictions on fungicide use, particularly in the European Union, and associated human health and environmental concerns (Calvert et al., 2008; Hinckley and Matson, 2011; Raanan et al., 2017; Bastos et al., 2021). SIGS may provide a novel strategy for *B. hordei* management if highly efficient dsRNA molecules are identified and developed further for practical application.

A number of SIGS experiments applied synthetic dsRNAs that were bioengineered to silence one or more components of the RNAi machinery in the target crop pathogenic fungi (Koch et al., 2016; Wang M. et al., 2016; Werner et al., 2020). Unlike previous studies, which primarily utilized long or short fragments of pathogen-derived mRNA to generate siRNAs for SIGS, our work introduces the use of plant microRNA (miRNA) mimics as direct agents of gene silencing. The expected benefit of using mimics of endogenous plant miRNA is that they will reduce the expression of their natural cross-kingdom targets. In this work, we tested for the first time the cross-kingdom gene silencing efficacy of some plant miRNA mimics for the control of *B. hordei* through SIGS. The miRNAs are short, typically 17-28-nucleotide-long single-stranded RNA molecules that regulate gene expression by targeting mRNA (Mann et al., 2023). Application of miRNAs in SIGS is still an unexplored research direction. We selected miRNAs that were shown in previous studies to be overexpressed in plants under biotic and abiotic stress conditions (Chen C. et al., 2025; Liu et al., 2022; Luo et al., 2024; Ulu et al., 2025; Xiao et al., 2017; Yang et al., 2022). Previous works have also demonstrated that barley can absorb exogenous dsRNA and process it to combat fungal pathogens (Gaffar et al., 2019; Schlemmer et al., 2022; Sundararajan et al., 2025). *Blumeria graminis*, a close relative of *B. hordei*, can also absorb exogenous dsRNA (Zhang et al., 2025). The main goal of our study was to identify for the first time plant miRNA mimics that reduce *B. hordei* infections through SIGS.

## 2 Materials and methods

### 2.1 Selection of plant-derived miRNAs based on literature and subsequent *in silico* analyses

First, three plant-derived miRNA families, miR167, miR444 and miR5048, were pre-selected based on their roles in plant-pathogen interactions, as reported in the literature (**Table 1**). As a second step, their sequences were used to search for their targets in the *B. hordei* genome (accession number: GCA_000151065.3) using psRNATarget tool with Scoring Schema V2 (Dai et al., 2018). To make the results more robust to determine whether the predicted miRNA targets are conserved across powdery mildew species (suggesting functional importance), the same *in silico* analyses were carried out in the genomes of the following other powdery mildew species, as well: *Erysiphe necator*, *Golovinomyces cichoracearum*, *Podosphaera aphanis* and *B. graminis*. Genome accession numbers are shown in **Table 2**.

**Table 1:** Roles of the selected plant-derived miRNA families in plant–pathogen interactions, as reported in the literature.

miRNA family	Roles in plant-pathogen interactions
miR167	Auxin signalling, suppression of pathogen proliferation (Zhang et al., 2011; Chen X. et al., 2025) Elevated expression during bacterial infections of cassava; virus infections of rice and maize; and fungal infection of rice (Zhang et al., 2011; Perez-Quintero et al., 2012; Li et al., 2014; Yang et al., 2016; Zhou et al., 2016; Liu X. et al., 2021) Components of the potato PVY virus (Iqbal et al., 2016) Trigger salicylic acid-dependent defense responses in *Arabidopsis* against bacterial infection (Caruana et al., 2020) Expression increased upon powdery mildew infection (Xin et al., 2010) Modulate the balance between plant growth and immune defense (Liu X. et al., 2021; Luo et al., 2024)
miR444	Regulation of transcription factors (MADS-box) which downregulate RNA-dependent RNA polymerase 1 (RDR1) (Wang H. et al., 2016; Yang et al., 2022; Chen X. et al., 2025) Induced expression during powdery mildew, leaf rust, rice stripe virus and brown planthopper attack in wheat and rice (Xin et al., 2010; Gupta et al., 2012; Kumar et al., 2014; Yang et al., 2016; Wu et al., 2017) Serve as a molecular switch between growth and immunity (Xiao et al., 2017)
miR5048	Confirmed targets are receptor-like kinase (RLK) gene *TaNAK1*, Cytosolic Ascorbate Peroxidase 6 (APX6), Azoospermia Factor 1 (AZF1), Mitogen-Activated Protein Kinase Kinase Kinase 17 (MAPKKK17), and Cytokinin Dehydrogenase (CKX5) (Wu et al., 2022; Yang et al., 2022) Predicted targets include nucleoprotein TPR, Casein Protein Kinase 2 (CK2), and Vesicle-Associated Membrane Protein 7 (VAMP7) (Ulu et al., 2025)

**Table 2:** Predicted targets of the four selected plant-derived miRNAs across different powdery mildew species inferred from psRNATarget tool and biological function analyses.

Powdery mildew species and genome acc. nos.	miR167c targets	miR444b targets	miR5048a targets	miR5048b targets
*Blumeria hordei* GCA_000151065.3	PQ loop repeat protein Serine/threonine-protein kinase Sgk2 60S ribosomal protein L7	Importin beta-1 subunit Mannitol-1-phosphate 5-dehydrogenase CSEP0284 effector protein ZIP metal ion transporter SAGA complex component (Sgf73) *Bh*-specific protein	Cyclin-dependent protein kinase Ssn3 Protein kinase (Gcn2) Serine/threonine protein kinase/sid2 DNA polymerase alpha catalytic subunit A NADH-ubiquinone oxidoreductase 29.9 kDa subunit DDHD domain-containing protein ADAM family of metalloprotease ADM-B Kinase domain containing protein/protein kinase Yak1 SANT domain-containing protein 2 P-loop containing nucleoside triphosphate hydrolase	Cyclin-dependent protein kinase Ssn3 Protein kinase (Gcn2) Serine/threonine protein kinase/sid2 RRP12-like protein Eukaryotic translation initiation factor 3 subunit M (eIF3m) Calcofluor white hypersensitive protein TBC domain-containing protein
*Erysiphe necator* GCA_000798715.1		Ph domain protein 60S ribosomal protein F-box wd repeat-containing protein Thiamine pyrophospho-kinase Oxysterol-binding protein Ribonuclease H-like domain	Meiotic mRNA stability protein kinase SSN3 protein Protein kinase Phosphatidyl-inositol 3 enzyme Serine-threonine protein kinase Nucleolar ATPase protein Cytochrome b561 ferric reductase transmembrane	Meiotic mRNA stability protein kinase SSN3 protein Protein kinase Tbc domain-containing protein
*Golovinomyces cichoracearum* GCA_003611235.1		Ras-like protein 2 Pre-mRNA-splicing factor cwc24	Serine/threonine-protein kinase Ssn3 Protein phosphatase PP2A regulatory subunit B Serine/threonine-protein kinase gad8 GRIP domain-containing protein Serine threonine protein kinase Phd-finger domain-containing protein Dipeptidyl-peptidase 5 Vacuolar protein sorting-associated protein 27	Serine/threonine-protein kinase Ssn3 Protein phosphatase PP2A regulatory subunit B Protein kinase GRIP domain-containing protein RNase p rpr2 rpp21 snm1 subunit domain-containing protein
*Blumeria graminis* f. sp. *tritici* (GCA_000418435.1)	Serine/threonine-protein kinase Sgk2 PQ-loop repeat-containing protein	Karyopherin beta Acetyl-CoA carboxylase biotin F-box/WD repeat-containing protein 7 ZIP metal ion transporter *Bgt*-specific protein Phosphoinositide PI45P(2) binding protein	Cyclin-dependent protein kinase Protein kinase Autophagy-related protein 29 Cobalamin-independent methionine synthase	Cyclin-dependent protein kinase Autophagy-related protein 29 Cobalamin-independent methionine synthase Eukaryotic translation initiation factor Catalytic subunit of the main cell cycle cyclin-dependent kinase (CDK)
*Podosphaera aphanis* GCA_022627015.2	PQ-loop repeat-containing protein Enoyl-[acyl-carrier-protein] reductase, mitochondrial	Copper resistance protein Phospholipase D1 Ribonuclease H-like domain Tkp3 protein Zinc knuckle domain protein Elongator complex protein 1 Bah domain-containing protein TATA-binding protein-associated factor Retrovirus-related Pol polyprotein from transposon TNT 1-94 Protein phosphatase PP2A regulatory subunit A Vacuolar protein sorting-associated protein 8-like protein	Protein kinase Serine/threonine-protein kinase Mitogen-activated protein kinase eIF-2-alpha kinase Serine/threonine-protein kinase Nek11	Protein kinase Reverse transcriptase RNA-dependent DNA polymerase Mitochondrial chaperone Frataxin Acetyl-CoA synthetase-like protein

Gene Ontology (GO) analysis was performed to characterize the functional roles of the *in silico* identified genes/proteins using OmicsBox 3.5.3 (Götz et al., 2008). Based on literature searches (**Table 1**) and *in silico* analyses (**Table 2**), one variant was selected of each of the miR167 and miR444 families; and two variants of the miR5048 family from the miRbase (Kozomara et al., 2019). The sequences of the selected miRNA variants – tested in the proof-of-concept experiment described below – were as follows:

miR167c: CTGAAGCTGCCAGCATGATCTG
miR444b: TTTTCTTGCAAGTTGTGCAGT
miR5048a: TTTGCAGGTTTTAGGTCTAAGT
miR5048b: TATTTGCAGGTTTTAGGTCTAA

### 2.2 Plant and fungal materials

A proof-of-concept experiment was carried out with barley cv. Stirling leaf segments placed in 90 mm diameter plates on 1% benzimidazole agar. Plates with leaf segments were kept in a Conviron GEN1000 plant growth chamber (Winnipeg, MB, Canada) at 20°C, 70% relative humidity, and 12 h daily illumination for the duration of the experiments. To produce powdery mildew-free leaf segments, barley cv. Stirling was grown from seeds in pots in an experimental glasshouse, in BugDorm^®^ cages with very fine mesh (MegaView Science Ltd., Taiwan) as described before (Kelly et al., 2021, 2025). Pots were watered through the mesh, without opening the cages, until plants were 15–18 cm tall, then taken to the laboratory where cages were opened and the first leaves of each plant were excised in a laminar flow cabinet. The middle parts of the excised leaves were cut into 4-cm-long segments, which were placed on benzimidazole agar and immediately sprayed with dsRNAs and control treatments as described below. Twelve hours following spray treatments, all leaf segments were inoculated with *B. hordei* as detailed below. The *B. hordei* inoculum was maintained on potted barley cv. Stirling kept in BugDorm^®^ cages with very fine mesh and watered without opening the cages.

### 2.3 *In vitro* synthesis of dsRNA molecules and SIGS experiment

To synthesize double-stranded versions – i.e., miRNA duplexes – of the selected four plant miRNAs (miR167c, miR444b, miR5048a, and miR5048b), the Silencer^®^ siRNA Construction Kit (Thermo Fisher Scientific Waltham, MA, USA) was used according to the manufacturer’s protocol. We have also synthesized a miRNA duplex using the positive control supplied with the Silencer^®^ Kit after verifying with the psRNA Target tool that its sequence (GTATGACAACAGCCTCAAGTT) has no predicted targets in *B. hordei*. This miRNA duplex was used as a non-target dsRNA control – i.e., a negative control – in all SIGS experiments to account for any sequence-independent effects of dsRNA application, and was referred to as the ‘kit control’ in this work. Sense and antisense DNA oligonucleotide templates were designed by adding 5′-CCTGTCTC-3′ sequences complementary to T7 promoter primers on 3′ side of miRNAs and their antisenses, which were subsequently sent to Macrogen Inc. (Seoul, Korea) for synthesis. The synthesized sense and antisense DNA nucleotide templates were modified to incorporate T7 promoter sequences at their 5′ ends, following the protocol provided with the kit. Sense and antisense primers containing T7 promoter sequences at their 5′ ends were used for *in vitro* synthesis of miRNA-derived dsRNAs via T7 RNA polymerase. The resulting dsRNAs of sense and antisense miRNA primers were then combined and hybridized according to the protocol provided with the kit. The synthesized dsRNAs were digested by DNase and RNase to remove non-miRNA sequences and purified according to the kit protocol. A small aliquot of the cleaned miRNA mimics was analyzed on a 2% agarose gel to confirm successful synthesis and verify the expected size. The concentration of miRNA mimics was quantified using a DS11 spectrophotometer (DeNovix, Wilmington, USA), and the purified dsRNA was subsequently diluted to 100 ng/μL for application onto barley leaves placed on 1% benzimidazole agar.

A total of 21 plates, 90 mm diameter, each containing six barley leaf segments on 1% benzimidazole agar, were treated each with 1 mL of one of the following materials: (i) either one of the four dsRNA variants of the synthetic miRNA duplexes; (ii) the mixture of the four dsRNA mimics; (iii) the kit control applied as a negative control as explained above; and (iv) DNase/RNase-free deionized water, used as another type of negative control. Plastic spray bottles, 10 mL, containing each three mL of one of the materials listed above, were used for SIGS in a biosafety cabinet. Three plates were used for each of the four synthetic miRNA duplexes; three for the miRNA duplex mixture; three for the kit control; and three for the treatment with DNase/RNase-free deionized water. The plates were then left open to allow the leaf segments to dry inside the cabinet. Twelve hours after the spray treatments, the cabinet fan was switched off, and a cloud of *B. hordei* conidia was generated above the open plates to ensure uniform inoculation of the leaf segments with powdery mildew. The *B. hordei* spore cloud was produced by gently blowing six-day-old *B. hordei* colonies on barley leaves that were freshly collected from potted barley plants kept in an experimental glasshouse. Inoculated plates were then transferred to a Conviron GEN1000 growth chamber. At 2.5 days post infection (dpi), two leaf segments from each plate were collected to determine the micro-colony index as described in section 2.4. At six dpi, another two leaves per plate were harvested for DNA extraction and subsequent qPCR analysis. Finally, at eight dpi the remaining two leaves from each plate were microscopically evaluated for visual symptoms of *B. hordei* infection. The experiment was performed twice.

### 2.4 Aniline blue staining to calculate the *B. hordei* micro-colony index

Barley leaf segments inoculated with *B. hordei* were collected at 2.5 dpi and placed in 15 mL centrifuge tubes. Ten mL ethanol was added to each tube, and samples were incubated overnight at room temperature to remove chlorophyll from the leaf tissues. The cleared leaf segments were stained with 0.01% Aniline Blue staining solution, prepared by dissolving aniline blue powder (Sigma-Aldrich) in 150 mM KH_2_PO_4_ at room temperature. After staining, the excess dye solution was discarded, and the leaves were examined under a light microscope. As described by Both et al. (2005), at 2.5 dpi those germinated *B. hordei* conidia that have already penetrated the epidermal cells of barley leaves and formed haustoria develop micro−colonies on the leaf surface, consisting of small, localized clusters of fungal hyphae. Other conidia remain ungerminated or do not penetrate epidermal cells following germination (Both et al., 2005). For each treatment, 500 *B. hordei* conidia were identified on the cleared barley tissues and grouped in the following categories: (a) non-germinated conidia; (b) conidia with germ tubes that did not develop into a micro-colony (i.e., did not infect the leaf tissues); and (c) conidia that produced a micro-colony. The micro-colony index was defined as the number of *B. hordei* micro-colonies per 100 conidia on each leaf segment. Statistical analysis was performed using an unpaired two-tailed Student’s t-test to compare control and treated samples. A p-value < 0.05 was considered statistically significant (*), and p < 0.01 was considered highly significant (**).

### 2.5 Pathogen relative DNA abundance quantification

For relative quantification of *B. hordei* DNA abundance between synthetic miRNA duplex−treated and control leaf segments, quantitative real−time PCRs (qPCRs) were performed using primers that amplify short fragments of the fungal nuclear ribosomal DNA (nrDNA). Although nrDNA regions are present in multiple copies in each genome, qPCRs based on nrDNA fragments could be used for comparative quantification purposes of the target fungal species in plant samples (Lievens et al., 2006). Leaf segments were collected at six dpi and DNA was extracted using a DNeasy Plant Mini Kit (QIAGEN, Germany). Primers used for qPCR were ITS1F (CTTGGTCATTTAGAGGAAGTAA) (Gardes and Bruns, 1993) and ITS2 (GCTGCGTTCTTCATCGATGC) (White et al., 1990). These primers amplify a 294 bp fragment of the ITS region of *B. hordei*. DNA samples were amplified using a CFX Opus 96 Real-Time PCR System (Bio-Rad, Hercules, CA, USA): initial denaturation at 95°C for 3 min, followed by 40 cycles of denaturation at 95°C for 30 sec, annealing at 55°C for 30 sec, and extension for 1 min at 72°C; and a final extension step at 72°C for 10 min. The reaction was carried out in 20 µL total volume using the iTaq Universal SYBR Green Supermix (Bio-Rad). Each reaction contained 10 µL iTaq Universal SYBR Green Supermix, 0.4 µM of each primer, and 200 ng template DNA as measured with a DS11 spectrophotometer. MCM7 primers were also developed for *B. hordei* qPCRs but were not useful in this work due to the low amount of powdery mildew DNA in the synthetic miRNA duplex-treated samples (data not shown).

qPCR data were analyzed to assess relative *B. hordei* ITS abundance levels in all samples taken from both repetitions of the experiment. Each reaction was performed in technical triplicates. As the same amount (200 ng) of total DNA was used, extracted from leaf segments infected with *B. hordei*, an internal plant gene control was not applied because plant DNA quantities were expected to differ in the synthetic miRNA duplex-sprayed leaf tissues compared to the controls. The relative quantification of the fungal DNA abundance was based on the *B. hordei* ITS fragment only in which ΔCt was calculated by subtracting the average of Ct values in samples sprayed with water and kit control from the average of the Ct values in synthetic miRNA duplex-sprayed samples. To obtain fold change values for relative fungal DNA abundance, 2−ΔCt was applied. qPCRs data were analyzed using Excel and statistical significance was determined using Student’s t-test. A p-value < 0.05 was considered statistically significant (*), and p < 0.01 was considered highly significant (**).

## 3 Results

### 3.1 Selection of plant-derived miRNAs for SIGS experiments

**Figure 1:**
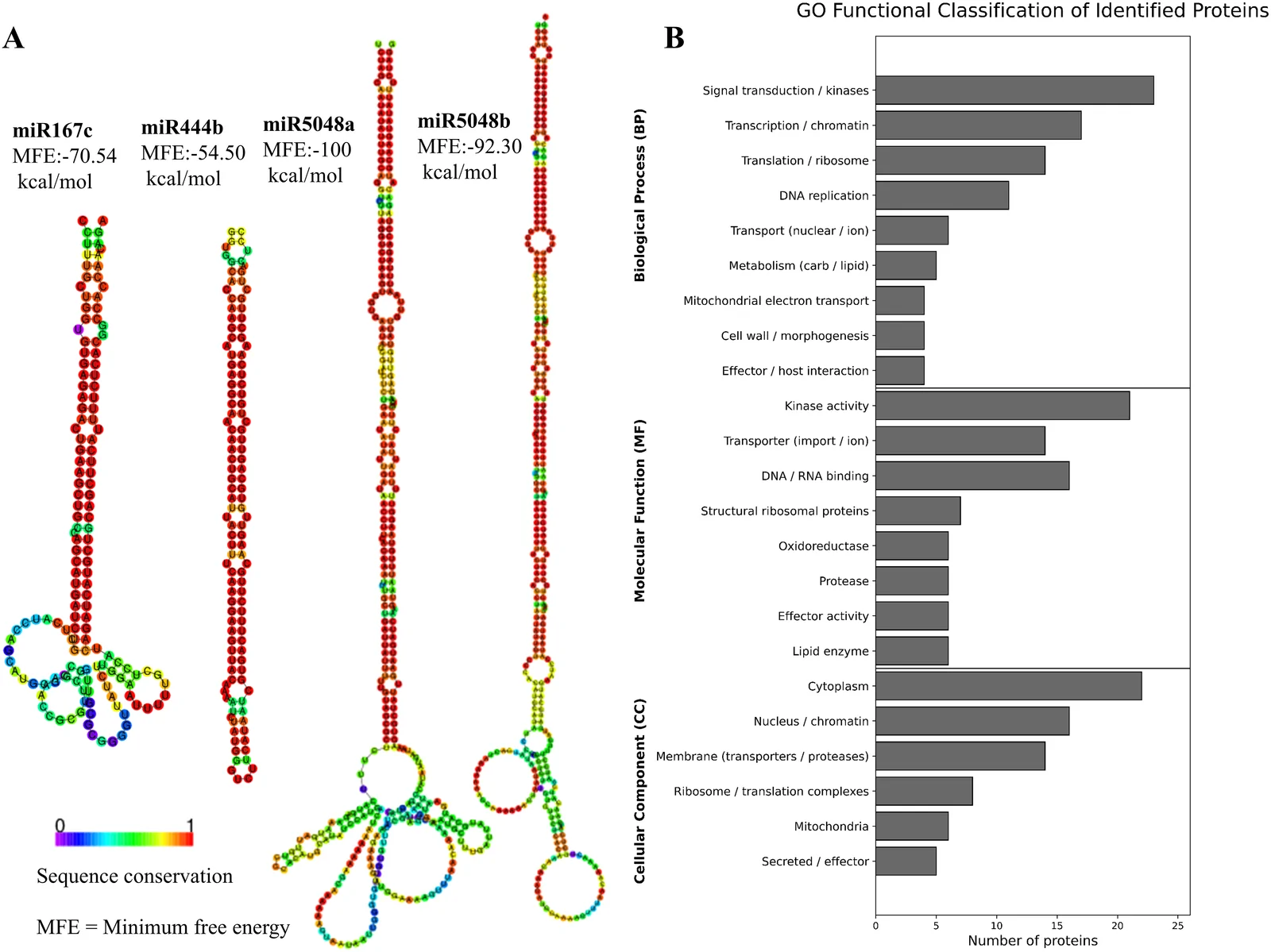
Predicted secondary structures of the four selected plant-derived miRNAs and Gene Ontology (GO) functional classification of miRNA target proteins. **(A)** Predicted secondary structures of the four selected miRNAs, highlighting their characteristic stem–loop conformations. The secondary structures of the precursor miRNA (pre-miRNA) molecules were predicted using the miRNAfold algorithm (Lorenz et al., 2011). These molecules form a characteristic stem–loop (hairpin) configuration, with the mature miRNA sequence on one arm of the hairpin. Base-pairing interactions are indicated by connecting lines, and mismatches or bulges are visible within the stem region. The minimum free energy (MFE) of the folded structures is also shown, reflecting the thermodynamic stability of the predicted hairpins. The positions of the miRNAs* (passenger strands) are opposite to the mature miRNA sequences. These structures satisfy the criteria for miRNA annotations, including a stable stem–loop, limited internal loops, and proper positioning of the mature miRNAs within the duplex region. **(B)** GO−Slim based functional categorization of the predicted miRNA target proteins revealed enriched terms across Biological Process, Molecular Function, and Cellular Component. Bar plots indicate the number of predicted proteins associated with each GO−Slim category.

Based on a literature review of plant-derived miRNAs in plant-pathogen interactions (**Table 1**) and predicted targets of some pre-selected miRNAs (**Table 2**), four miRNAs were selected for further studies. First, the sequences of the four selected variants (**Figure 1A**), were used for *in silico* predictions of their targets in the *B. hordei* genome GCA_000151065.3. More than one target *B. hordei* gene was identified for each of the selected miRNA variants (**Table 2**). GO functional classification of the predicted targets revealed that putative proteins associated with chromatin organization, transcriptional regulation, signalling, and metabolic processes may be impacted by the selected miRNAs (**Figure 1B**).

To predict whether the selected miRNA variants may target genes in other powdery mildews, as well, the same *in silico* analyses were also conducted using the genomes of the following species: *B. graminis*, *E. necator*, *G. cichoracearum*, and *P. aphanis*. As shown in **Table 2**, some of the predicted targets of the four miRNAs were different in the powdery mildew species included in our analysis. Interestingly, no predicted target was identified for miR167c in *E. necator and G. cichoracearum*, whereas the PQ loop repeat protein was targeted by miR167c in *B. hordei*, *B. graminis*, and *P. aphanis*. Protein kinases were targeted by miR5048a and miR5048b in all powdery mildews included in this study (**Table 2**). The predicted targets of miR444b were different in all powdery mildews (**Table 2**).

### 3.2 Production of duplexes of the selected miRNAs

**Figure 2:**
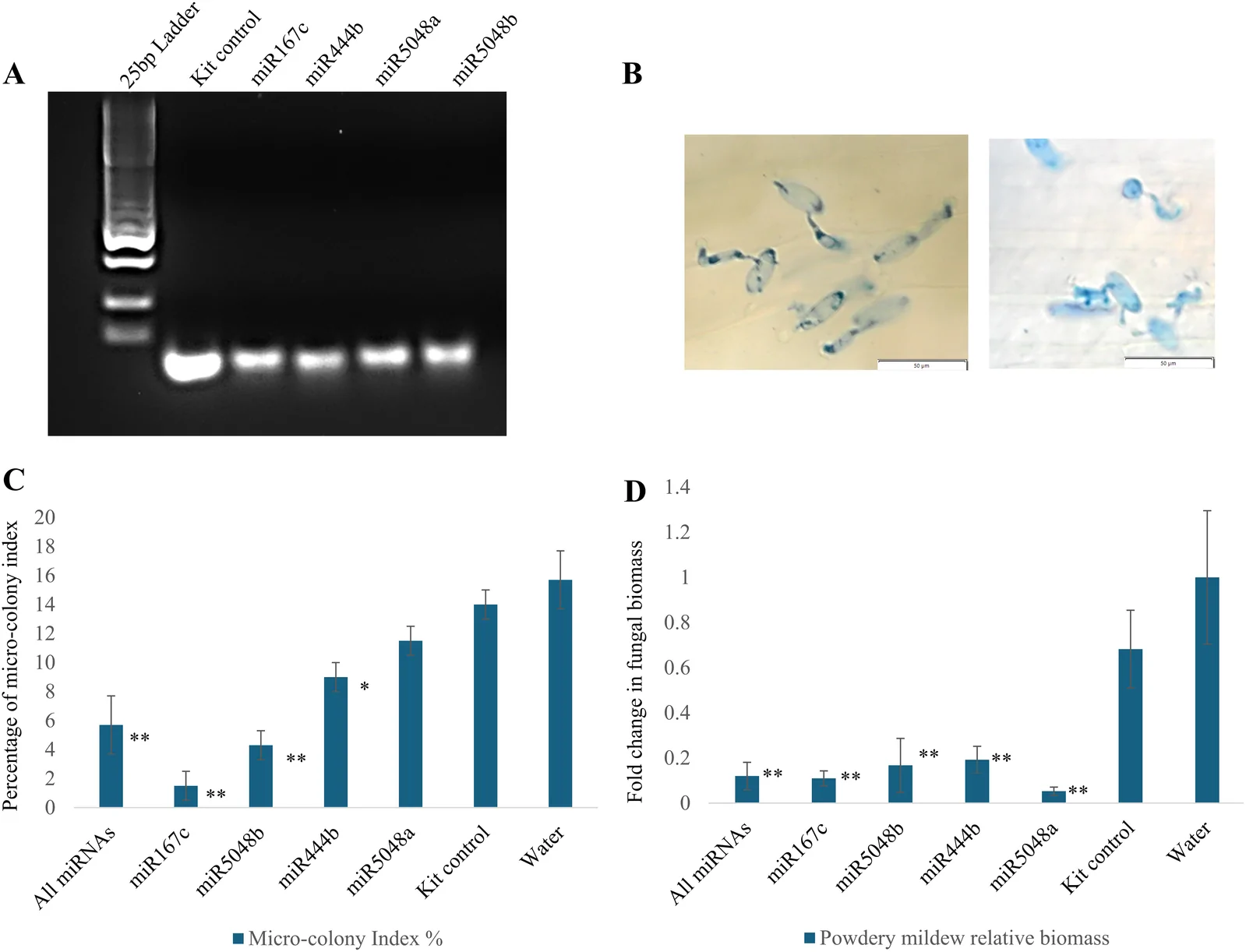
Synthesis of the four selected stress-responsive plant miRNA mimics and results of the Spray-Induced Gene Silencing (SIGS) experiments with barley leaf segments uniformly inoculated with conidia of *Blumeria hordei* 12 hours following SIGS. **(A)** Double-stranded RNAs (dsRNAs) corresponding to the selected miRNA mimics and the Silencer^®^ Kit control were synthesized *in vitro* and visualized by gel electrophoresis. All dsRNA samples produced distinct bands below 25 bp, confirming successful synthesis and the absence of overhanging sequences. **(B)** Application of stress-responsive plant miRNA mimics inhibited the formation of micro-colonies originating from germinated *B. hordei* spores. Left: germinated *B. hordei* conidia that failed to form micro-colonies on a barley leaf segment treated with a miRNA mimic, and cleared for microscopy. Right: *B. hordei* micro-colonies on a water-treated leaf segment, cleared for microscopy. **(C)** *B. hordei* micro-colony indices determined at 2.5 days post infection (dpi) on barley leaf segments treated with either one of the four selected miRNA mimics or their mixture, DNase/RNase-free deionized water, or the Silencer^®^ kit control applied as a negative control. **(D)** *B. hordei* DNA abundance determined at six dpi on barley leaf segments treated as in 2C. Asterisks denote statistical significance: *p < 0.05, **p < 0.01.

To test the potential anti-powdery mildew effect of the four selected miRNAs, duplexes of each miRNA mimics were synthesized *in vitro* using a Silencer^®^ kit. Each newly synthesized dsRNA product displayed a single distinct band below the 25 bp marker of HyperLadder™ 25bp (**Figure 2A**). This indicated the absence of any unwanted polynucleotides at either the 3´ or the 5´ ends of the 21 or 22 bp long miRNA mimics, which may have had an impact on the results obtained during the proof-of-concept experiment. dsRNA was also synthesized from the Silencer^®^ kit control and the product appeared as a single band below the 25 bp marker (**Figure 2A**).

### 3.3 Three out of four selected miRNA mimics applied through SIGS reduced the *B. hordei* micro-colony index

The double-stranded copies of the selected miRNA mimics were each sprayed individually, as well as in combination, as a mixture, onto barley leaf segments maintained on benzimidazole agar. Control leaf segments were sprayed with either sterile DNase/RNase-free deionized water or the kit control; these treatments were referred to as the water and the kit control, respectively. All leaf segments were uniformly inoculated with *B. hordei* conidia 12 hours after spraying. At 2.5 dpi, the *B. hordei* micro-colony index was determined for each treatment using light microscopy.

Under the microscope, *B. hordei* conidia that successfully produced micro-colonies at 2.5 dpi were readily distinguished from those that failed to do so. On miRNA mimic-treated barley tissues, many germinated conidia were unable to establish micro-colonies, whereas on control leaf segments most germinated conidia developed colony initials (**Figure 2B**). The micro-colony index was 15.7% on water-sprayed leaf segments; 14% for the kit control; 5.7% for the mixture of the miRNA mimics; 1.5% for miR167c; and 4.3%, 9%, and 11.5% for miR5048b, miR444b, and miR5048a, respectively (**Figure 2C**). Following SIGS with miRNA mimics mixture, miR167c and miR5048b, the micro-colony indices were significantly lower at p<0.01 compared to the water control. SIGS with miR444b resulted in micro-colony indices that were significantly lower compared to the control at p<0.05. The treatments with miR5048a did not produce significantly lower micro-colony index values compared to the water or the kit control (**Figure 2C**).

**Figure 3:**
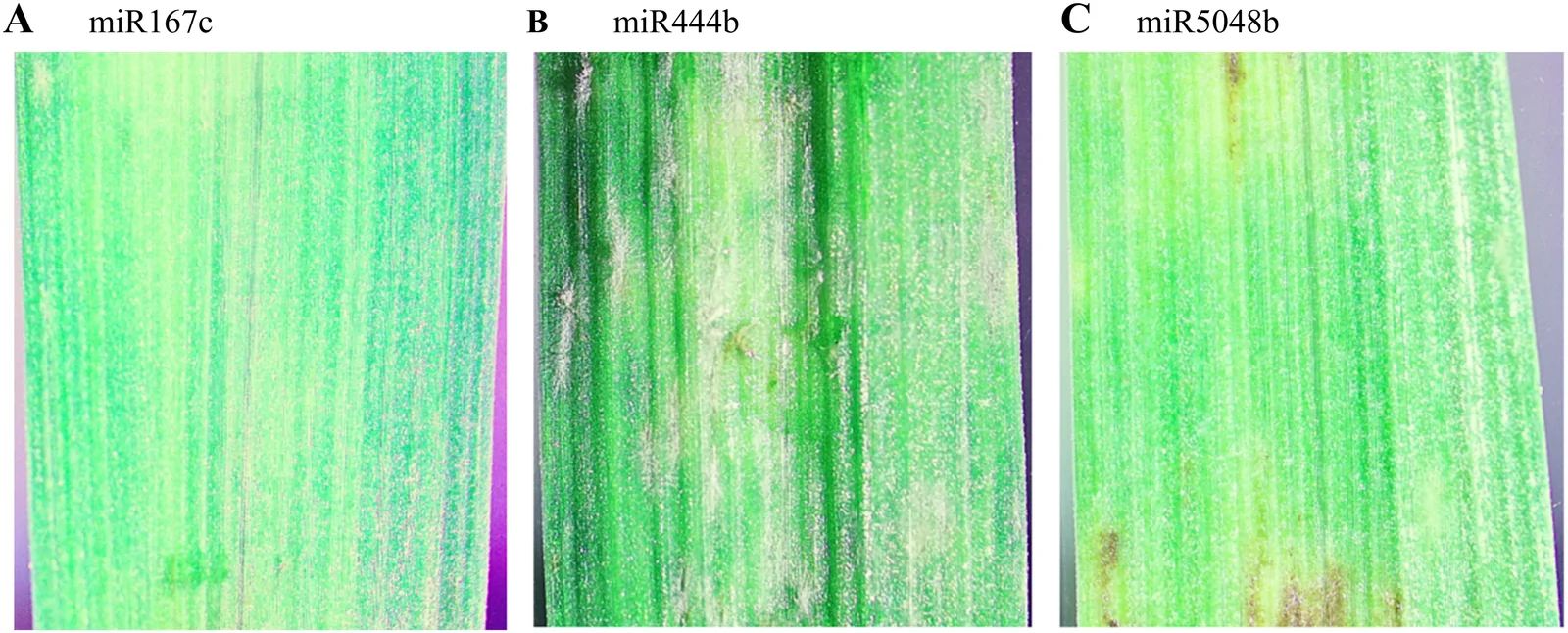
Impact of Spray-Induced Gene Silencing treatments of barley leaf segments uniformly inoculated with conidia of *Blumeria hordei* 12 hours post treatment. Images were taken eight days post-inoculation. **(A)** Treatment with miR167c. **(B)** Treatment with miR444b. **(C)** Treatment with miR5048b.

**Figure 4:**
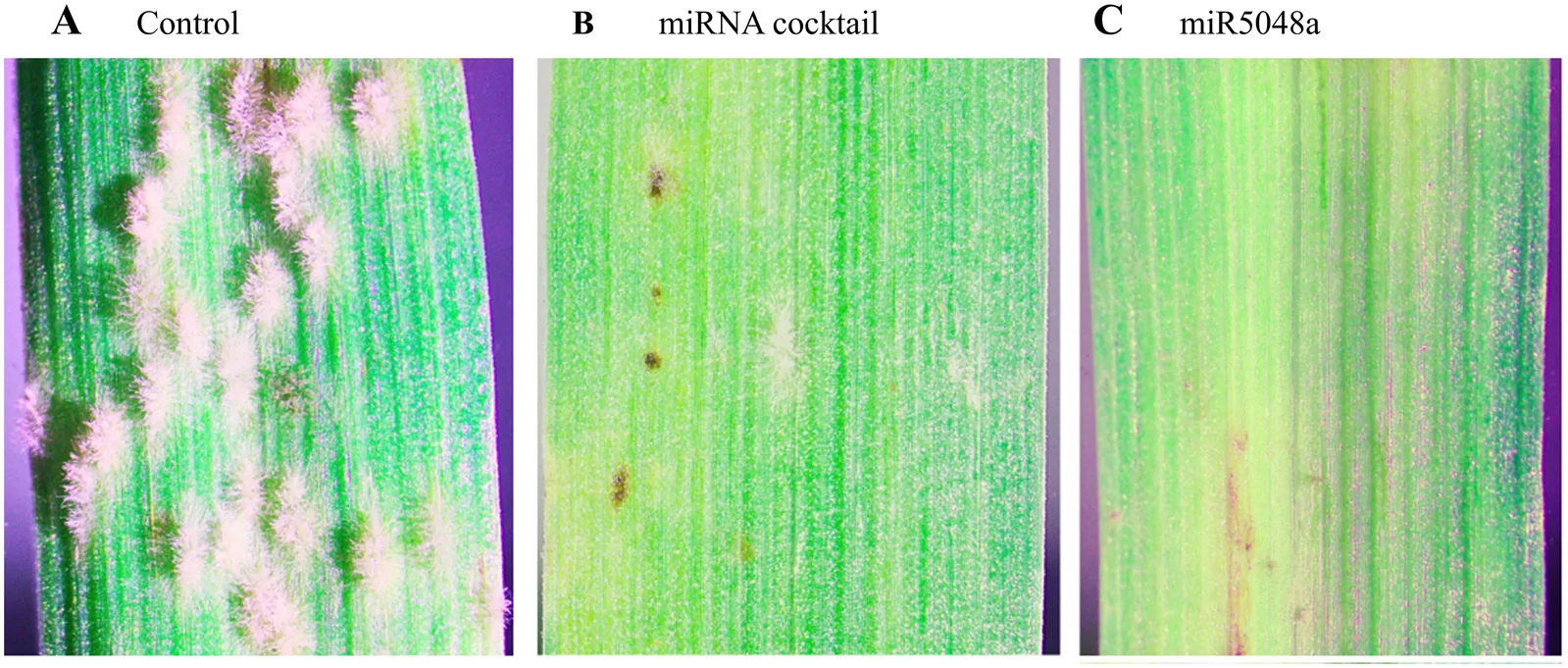
Impact of control and Spray-Induced Gene Silencing treatments of barley leaf segments uniformly inoculated with conidia of *Blumeria hordei* 12 hours post treatment. Images were taken eight days post-inoculation. **(A)** Control treatment with DNase/RNase-free deionized water. **(B)** Treatment with a mixture of all four selected miRNA mimics. **(C)** Treatment with miR5048a. This treatment resulted in the most evident protective effect, with minimal signs of disease symptoms and largely preserved leaf tissue integrity.

For the miR167c-treated samples, the micro-colony index was 10-fold and 9-fold lower than those of the water- and kit control-sprayed leaf segments, respectively, at 8 dpi (**Figure 2C**). These values were supported by visual assessments, as symptoms developing on miR167c-treated leaf segments (**Figure 3A**) were markedly less severe than those observed on the water control (**Figure 4A**). However, micro-colony index values did not fully correspond with symptom development following miR444b and miR5048a treatments. In these cases, the micro-colony index was 1.7-fold and 1.14-fold lower than that of the water-sprayed leaf segments, respectively (**Figure 2C**), and 1.5-fold and 1.2-fold lower than that of the kit control-treated leaves, respectively. Despite these micro-colony index values, visual symptoms were comparable to those observed in miR167c-treated leaf segments (**Figures 3B, C**). In contrast, miR5048b treatment resulted in micro-colony indices that were 3.6-fold and 3.2-fold lower than those of the water-sprayed and kit control-treated leaves, respectively (**Figure 2C**). These reductions (**Figure 2D**) were consistent with the observed disease symptoms.

Although it was anticipated that leaf segments treated with the mixture of the miRNA mimics would exhibit a stronger reaction, this was not observed. Leaf segments sprayed with the miRNA mimics mixture showed a 2.7-fold reduction in powdery mildew micro-colony index compared to water-sprayed leaves, and a 2.5-fold reduction compared to kit control (**Figure 2C**) aligned with the visual symptoms (**Figure 4B**).

### 3.4 All four selected miRNA mimics applied through SIGS reduced the *B. hordei* DNA biomass on the inoculated barley leaf segments

The *B. hordei* DNA abundance was determined at six dpi and was significantly (p< 0.05) reduced in all barley tissues treated with each of the four miRNA mimics, and their mixture, as well, compared to the controls (**Figure 2D**). While relative *B. hordei* DNA abundance values generally correlated with the visual symptoms observed on the leaf segments at eight dpi (**Figures 3**, **4**), the qPCR results did not fully correlate with the micro-colony indices determined at 2.5 dpi.

The spray with the kit control resulted in 1.23 times lower *B. hordei* DNA abundance values compared to the water control, which aligned with the micro-colony indices (**Figure 2C**) and the visual symptoms at eight dpi (**Figures 3**, **4**). Although not significantly different from the other miRNA mimics treatments, including the mixture, it should be noted that miR5048a caused the largest decrease in *B. hordei* DNA abundance, which was 16-fold lower compared to the water control and 13-fold lower compared to the kit control at six dpi. In contrast, the micro-colony index determined in miR5048a-treated leaf tissues at 2.5 dpi was not significantly different from the controls (**Figure 2C**). In the case of the mimics of miRNA167c, miRNA5048b, and miRNA444b, both micro-colony indices and *B. hordei* DNA abundance values were significantly lower than the controls (**Figures 2C, D**). The *B. hordei* relative DNA abundance values aligned with the visual symptoms observed at 8 dpi (**Figures 3**, **4**).

## 4 Discussion

This is the first study to show that plant-derived, stress-responsive miRNA mimics reduce infection caused by fungal pathogens when applied through SIGS. Previous works primarily utilized long or short fragments of pathogen-derived mRNA to generate siRNAs for SIGS (Gaffar et al., 2019; Koch et al., 2016, 2019, 2020; Schlemmer et al., 2022; Zhang et al., 2025). Thus, these approaches for SIGS did not explore the natural cross kingdom targets but only the selected target mRNA of the pathogen. Our work introduced the use of mimics of endogenous plant-derived miRNAs to target pathogens through SIGS which explore the natural targets of these miRNA mimics in pathogens. It has already been revealed that *B. hordei* contains the RNAi machinery and produces diverse siRNAs (Kusch et al., 2018; Hunt et al., 2019) – therefore, the pathogen is capable to process dsRNA into active siRNAs, which can downregulate the pathogen’s target genes.

This study also represents the first application of SIGS against barley powdery mildew. To date, only one SIGS study has focused on cereal powdery mildew control, targeting *B. graminis* infecting wheat and in this study the approach of utilizing long or short fragments of pathogen-derived mRNA to generate siRNAs has been followed (Zhang et al., 2025). Our approach of exploring cross-kingdom targets of endogenous plant-derived miRNAs is inspired by discoveries related to signaling pathways between plants and their pathogens that are apparently mediated by cross-kingdom miRNAs. It appears that these molecules are involved in plant resistance against pathogens in nature but are never used in SIGS (Zhang et al., 2016; Shahid et al., 2018; Jannesar et al., 2026).

For a successful SIGS experiment with plants and their fungal pathogens, it is important to know if the plant and fungus can absorb the environmental dsRNA, then dsRNA can be converted into single stranded siRNAs which can be transported cross kingdom. These aspects have already been investigated in the barley-*Blumeria* pathosystem (Schlemmer et al., 2022; Gaffar et al., 2019; Zhang et al., 2025) that is why we proceeded with the SIGS experiment. Similar uptake of dsRNA was observed in other fungal pathogens, as well, including *G. orontii* (McRae et al., 2023), *Fusarium graminearum* (Koch et al., 2016; Qiao et al., 2021), *B. cinerea* (Qiao et al., 2021), *Sclerotinia sclerotiorum* (Qiao et al., 2021; Ouyang et al., 2025) and *Phakopsora pachyrhizi* (Ouyang et al., 2025). Notably, silencing of the *B. graminis* actin gene resulted in abnormal appressoria formation and penetration defects, further supporting the potential of SIGS in powdery mildew control (Zhang et al., 2025). Instead of artificial siRNAs, here we tested a set of plant-derived miRNA mimics to test the concept they could be used through SIGS as a disease control option.

According to Zhang et al. (2025), *B. graminis* directly takes up environmental dsRNA, followed by processing through its endogenous RNA interference machinery. This model is consistent with the demonstrated effectiveness of both host-induced gene silencing (HIGS) and SIGS in powdery mildew fungi (Padilla-Roji et al., 2023). Foliar-applied dsRNA uptake by plant tissues and then their transfer into EVs was reported in barley-*B. hordei* interactions (Schlemmer et al., 2021, 2022). However, barley EVs that transfer siRNAs into the mycelia of the pathogen have not been reported yet. Nevertheless, the transport of internal siRNAs from plants to pathogens – but not foliar-applied dsRNA – was detected in a number of plant-pathogen interactions (Hou et al., 2019; Singla-Rastogi et al., 2019) including *B. cinerea* (Cai et al., 2018). Also, there is evidence for EV accumulation at *B. hordei* infection sites – and the EVs extracted from *Blumeria*-infected barley tissues contained canonical sRNAs from both barley and *Blumeria* (Thieron et al., 2024).

After applying synthetic duplexes of the selected miRNAs, the largest observed decrease in the powdery mildew micro-colony index was 10-fold, and the *B. hordei* DNA abundance decreased by up to 16-fold compared to the water control. Visual disease symptoms supported the impact of miRNA mimics on *B. hordei* infection levels. Interestingly, the mixture of the four miRNA mimics was less effective than some of the treatments with individual miRNA mimics; and leaf segments treated with the miRNA mimics mixture exhibited increased yellowing compared to the water and the kit control.

Our results indicate that the four selected stress-responsive plant miRNA mimics are highly effective in suppressing *B. hordei* infections. If these miRNA mimics are engineered, they may become useful in the plant protection practice. The results revealed two distinct categories of pathogen suppression. miR444b and miR5048a led to moderate reductions in *B. hordei* micro-colony formation (25%–49%). In contrast, treatments with the miRNA mimics mixture and miR167c and miR5048b resulted in a statistically significant reduction in *B. hordei* micro-colony index (60%–90%) compared to both water and kit control-sprayed leaves. Consistent with these findings, the relative *B. hordei* DNA abundance values were also significantly reduced by approximately 80%-90% in miRNA mimics-treated samples. Visual disease symptoms generally supported the reductions observed in both micro-colony index and relative fungal DNA abundance.

The selected miRNA mimics reduced *B. hordei* infection to levels comparable to or exceeding those reported in previous SIGS studies. For example, Zhang et al. (2025) achieved 26%–50% reduction in wheat powdery mildew severity at eight dpi when the *B. graminis actin* gene was targeted using a 246 bp dsRNA fragment. Downregulation of *F. graminearum CYP* genes using dsRNA constructs derived from *CYP* mRNA sequences achieved up to 75% reduction in fungal DNA in *CYP3*-targeted, dsRNA-sprayed barley leaves (Koch et al., 2016). Similarly, SIGS was used to target *CYP51* mRNA and 40-60% reduction was observed (McRae et al., 2023). When SIGS was employed to target virulence effector genes (*PpAE1*, *PpAE2*, and *PpAE3*) of soybean rust caused by *P. pachyrhizi*, pathogen biomass was reduced by 64.8% (Ouyang et al., 2025). SIGS of *Rhizoctonia solani* polygalacturonase genes resulted in a 40%–60% reduction in fungal biomass (Qiao et al., 2021). In *Verticillium dahliae*, dsRNAs targeting *Vd-DCTN1* + *SAC1* and *Vd-DCL1* + *DCL2* led to 60%–70% reductions and for *B. cinerea*, dsRNAs targeting *Bc-VPS51* + *DCTN1* + *SAC1* or *Bc-DCL1/2* significantly reduced lesion size (Cai et al., 2018).

This study indicated that the designed miRNA mimics are effective against *B. hordei*. Future RT-qPCR or transcriptomic analyses are needed to confirm that the predicted fungal target genes are downregulated following SIGS. These experiments will provide additional evidence that disease suppression is linked to silencing of specific *B. hordei* genes.

Here, we tested a miRNA mimics-based SIGS strategy in a detached barley leaf assay under controlled laboratory conditions as a proof-of-concept experiment. While this experiment reliably measured antifungal activity, a limitation is that it cannot be considered as evidence for whole-plant activity and systemic response to treatments. Future glasshouse and field studies with whole plants are needed to examine the potential of the four miRNAs for sustainable powdery mildew control. There is evidence that both siRNAs and dsRNAs applied through SIGS are translocated through the vascular system of barley in a systemic way (Biedenkopf et al., 2020).

SIGS has emerged as a promising RNA-based technology for controlling plant diseases, but several challenges remain (Mann et al., 2023; He et al., 2024). The efficiency of RNA uptake differs between fungal species (Chen et al., 2023), and the RNA uptake mechanisms of obligate biotrophic fungi like *B. hordei* have not been fully elucidated (Padilla-Roji et al., 2023). Also, RNA-based protection tends to be short-lived and can be affected by environmental conditions, the target gene chosen, and the formulation of the RNA (Chen et al., 2023; He et al., 2024). More research is needed to improve RNA delivery and stability. One of the main challenges is maintaining RNA stability after it is sprayed onto leaves (He et al., 2024). Naked RNA can degrade quickly due to sunlight, rain, and enzymes on the leaf surface, reducing its effectiveness (He et al., 2024). Future research should explore new ways to protect and deliver RNA, such as using nanoparticles or other carriers, to improve its stability, uptake, and long-term efficacy in the field (Chen et al., 2023; Al Mamun et al., 2025).

Our approach represents a rapid, efficient, and non-transgenic strategy to decipher the functional roles of plant-derived sRNAs in plant–pathogen interactions. The classical approach requires stable transgenic lines for such studies, which is often time-consuming, technically challenging, and limited to species amenable to transformation. SIGS offers a practical and versatile alternative to traditional transgenic overexpression systems, particularly for studies requiring timely functional validation or involving recalcitrant plant species.

## Data Availability

The original contributions presented in the study are included in the article. Further inquiries can be directed to the corresponding author.
